# The Rise of IGFBP4 in People with Obstructive Sleep Apnea and Multilevel Sleep Surgery Recovers Its Basal Levels

**DOI:** 10.1155/2021/1219593

**Published:** 2021-10-04

**Authors:** Abdulmohsen Alterki, Eman Al Shawaf, Irina Al-Khairi, Preethi Cherian, Devarajan Sriraman, Maha Hammad, Thangavel A. Thanaraj, Mahmoud A. K. Ebrahim, Fahd Al-Mulla, Mohamed Abu-Farha, Jehad Abubaker

**Affiliations:** ^1^Department of Otolaryngology Head & Neck Surgery, Zain and Al Sabah Hospitals and Dasman Diabetes Institute, 15462, Kuwait; ^2^Department of Biochemistry and Molecular Biology, Dasman Diabetes Institute, 15462, Kuwait; ^3^Special Service Facility Department, Dasman Diabetes Institute, 15462, Kuwait; ^4^Department of Genetics and Bioinformatic, Dasman Diabetes Institute, 15462, Kuwait

## Abstract

IGFBP4 is the smallest member of the insulin-like growth factor binding protein family (IGFBP). It is a hepatic protein that plays a role in modulating the activity and bioavailability of IGF-I. The expression of IGFBP4 was found to increase under conditions of hypoxia. Obstructive sleep apnea (OSA) is a common disorder, characterized by cyclic episodes of intermittent hypoxia and fragmented sleep. Our aim was to quantify levels of circulating IGFBP1, IGFBP2, IGFBP3, IGFBP4, and IGFBP7 in fasting plasma samples of 69 Kuwaiti participants and explore its correlation with indices of OSA. The quantification was performed using multiplexing assay. The study involved 28 controls and 41 patients with OSA. Levels of circulating IGFBP4 were significantly higher in people with OSA (289.74 ± 23.30 ng/ml) compared to the control group (217.60 ± 21.74 ng/ml, *p* = 0.028). The increase in IGFBP4 correlated significantly and positively with AHI (*r* = .574, *p* = .01) and AI (*r* = .794, *p* = .001) in people with moderate and severe OSA. There was a significant decline in circulating IGFBP4 after 3 months of surgery (225.89 ± 18.16 ng/ml, *p* = 0.012). This was accompanied by a prominent improvement in OSA (AHI 8.97 ± 2.37 events/h, *p* = 0.001). In this study, our data showed a significant increase in circulating IGFBP4 in people with OSA. We also report a significant positive correlation between IGFBP4 and indices of OSA at baseline, which suggests IGFBP4 as a potential diagnostic biomarker for OSA. There was a significant improvement in OSA after 3 months of surgical intervention, which concurred with a significant decline in IGFBP4 levels. Altogether, the detected change suggests a potential link between IGFBP4 and OSA or an OSA-related factor, whereby OSA might play a role in triggering the induction of IGFBP4 expression.

## 1. Introduction

Obstructive sleep apnea (OSA) is a chronic sleep disorder that features fragmented sleep. It is characterized by having repeated episodes of airflow cessation (apnea) or airflow reduction (hypopnea), which leads to intermittent hypoxia (i.e., a decline in oxyhemoglobin saturation), interrupted sleep, and augmented heart rate oscillations, intrathoracic pressure, and hypertension [1, 2]. The chronic presence of these disruptions activates various pathological mechanisms that would elicit vascular damage and might play a role in the development of cardiovascular morbidities [1, 3]. OSA is a common disorder that is mostly prevalent among people with obesity [1] and has been associated with diabetes and cardiovascular disease (CVD) [4].

OSA treatment options involve lifestyle modifications, weight reduction, and the use of surgical procedures or external therapies that help keeping an open airway during sleep. Continuous positive airway pressure (CPAP) is the primary treatment as it was one of the first discovered and yielded the fastest recovery rates; however, its effectiveness depends on having a consistent use, and both compliance rates and consistency are highly compromised [5, 6], especially for those with minimal symptoms. Studies found weight loss to concur with a reduced apnea-hypopnea index (AHI) that indicates an abated severity and improved apnea symptoms in people with obesity and OSA [7, 8]. Weight-loss surgeries proved to be a better option for a rapid and efficient weight reduction. Therefore, in a meta-analysis protocol for bariatric surgery, the consideration of a surgical weight-loss procedure was strongly recommended for treating people with obesity and OSA [9]. Although the loss of excess weight per se is an important factor to relieve symptoms of OSA, ENT surgical intervention is one of the methods employed for OSA management that demonstrate being effective. These surgeries mitigate symptoms of OSA by enlarging and stabilizing the upper airway to reduce upper airway obstruction [10]. ENT procedures involve nasal, palate, tongue, and maxilla-mandibular surgeries [11]. Adenotonsillectomy and tonsillectomy are the most common procedures performed as an initial therapy in pediatric OSA, while nasal surgeries, uvulopalatopharyngoplasty, and tongue-based surgeries are the first-line OSA treatment in adults [12]. Nonetheless, OSA involves one or more upper airway levels. Thus, a multilevel sleep surgery (MLS) in a single-stage procedure has been developed as a surgical treatment method for OSA patients that require surgery, with a success rate reaching 60% [13, 14]. While postsurgery improvement is evident, the mechanisms/biochemical factors that are related to OSA and involved in the postsurgery improvement are yet to be identified.

The family of insulin-like growth factor binding proteins (IGFBPs) comprises a group of high-affinity binding proteins (IGFBP1–IGFBP6) [15]. An additional member is IGFBP-related protein-1 (IGFBP-rp1), which possesses up to 45% similarity to other IGFBPs and is better known as IGFBP7 [16]. Although highly similar, IGFBP7 differs in lacking the conserved c-terminus cysteines and having a reduced affinity for IGF-I but a strong affinity to insulin [17]. These proteins play a critical role in the GH/IGF system, by regulating the bioavailability and modulating the signaling of insulin-like growth factor (IGF) ligands [15]. The GH/IGF system and IGFBPs are influenced by sleep and sleep disordered breathing [18]. This suggests a potential involvement of IGFBPs in OSA or its related mechanisms. Members of the IGFBP family are involved in various cellular processes such as cell proliferation, differentiation, migration, and apoptosis, which explain their contribution to various physiological and pathological processes [15, 19].

The hepatic IGFBP1 is involved in various processes like insulin sensitivity [20], glucose regulation [20], and cardiovascular pathophysiology [20, 21]. The expression of IGFBP1 is raised at stressful conditions such as hypoxia and starvation to limit the activity of IGFs and consequently reduce the rate of growth and development [19]. Regulating cell growth and apoptosis was also reported with IGFBP3, which is the most abundant protein of the IGFBP family [22]. Low levels of circulating IGFBP3 have been associated with an increased CVD risk and higher mortality [23, 24]. In relation to OSA, the severity of sleep apnea (indicated by AHI score) correlated negatively with IGF-I levels, which was independent of BMI and age [18, 25]. The importance of IGFBP3 was emphasized by showing a link between decreased levels of circulating IGFBP3 and growth retardation in children with OSA [26]. IGFBP4, the smallest member of the IGFBPs, is thought to have a complex activity that is cell type and tissue-specific [27]. Studies have reported a critical role for IGFBP4 in vascular tissue physiology and pathophysiology, where it functioned as a regulator of smooth muscle cell proliferation [15, 27, 28]. This implicated a potential involvement of IGFBP4 in an OSA-induced effect.

This study is aimed at measuring circulating levels of IGFBPs in adult OSA patients and comparing them to control participants. We also investigated the association between IGFBPs and OSA indicators in adults to identify novel biomarkers. Additionally, we explore the use of surgical interventions as a treatment option and its effect on OSA and IGFBP levels.

## 2. Materials and Methods

### 2.1. Study Population and Design

The study involved a total of 69 participant; of these, 41 with OSA indicated by AHI > 5 events/h, and 28 were control defined by AHI < 5 events/h. Type I polysomnography (PSG) test was performed in level 1 sleep laboratory to determine the presence of sleep apnea. Participants were matched for age and body mass index (BMI). Inclusion criteria were those who underwent MLS and completed a preoperative and postoperative level 1 polysomnography (PSG), preoperative and postoperative Epworth Sleepiness Scale (ESS), and preoperative and postoperative blood metabolites, and we recorded their medical history and patient's data, such as BMI. The study excluded people with CVDs, diabetes, and a history of any major illness. Study approval was obtained by the Ethical Review Board of Dasman Diabetes Institute (DDI), and it was conducted in accordance with the ethical guideline outlined in the Declaration of Helsinki. Written consents were obtained from all participants prior to their enrollment in the study.

### 2.2. Biochemical and Anthropometric Measurements

Fasting blood samples were obtained from all participants. Plasma was extracted from blood samples collected in vacutainer (EDTA tubes), after 400 × g centrifugation for 10 min. Collected plasma samples were aliquoted and stored at -80°C until assayed. Blood pressure was measured by the Omron HEM-907XL digital sphygmomanometer. The final reading represented the average of three consecutive readings. Fasting blood glucose (FBG), serum total cholesterol (TC), low-density lipoprotein (LDL), high-density lipoprotein (HDL), and triglycerides (TG) were measured by Siemens Dimension RXL chemical analyzer (Diamond Diagnostics, Holliston, MA, USA). Hemoglobin A1c was quantified by the Variant™ device (Bio-Rad, Hercules, CA).

### 2.3. OSA Assessment and Surgery

The study population was diagnosed using Type I PSG test as detailed [29]. This test involves an overnight sleep in a sleep lab, during which biophysiological data was captured to formulate a comprehensive picture of participants' sleep architecture. Type I PSG includes monitoring blood oxygen levels, breathing patterns, brain activity (EEG), eye movement (EOG), heart rhythm (ECG), and skeletal muscle activity (EMG) during sleep. Data reflecting airflow and respiratory effort is used to calculate the apnea-hypopnea index (AHI). The apnea index (AI) indicated the number of events at which respiration is completely ceased for >10 sec/h, while hypopnea index (HI) reflected the number of partial airway obstruction events per hour. Diagnosis of OSA is based on participant's AHI score, where a score of >5 events/h of sleep is defined as an abnormal respiratory function during sleep. The presence of a high AHI score and its occurrence with excessive daytime sleepiness indicated a positive case of OSA. AHI score is used to evaluate the severity of OSA, with AHI score of 5 to 15 events/h reflected mild OSA, AHI of 15–30 events/h indicated a moderate level, and AHI score > 30 events/h defined a condition of sever OSA [30]. During sleep study (polysomnography) and while the patient is asleep, the oxygen saturation was measured using pulse oximetry. Pulse oximetry records oxygen saturation and heart rate levels of patients overnight while asleep. The average of pre- and postsurgery oxygen saturation (%) was used for analysis. All participants that underwent surgeries were carefully selected, and an individualized procedure was performed for each participant. All surgeries involved tonsillectomy in addition to other corrections such as the nasal cavity, nasopharynx, and/or hypopharynx depending on the cause of upper-airway collapse.

### 2.4. IGFBP Quantitative Assays

Levels of IGFBP1, IGFBP2, IGFBP3, IGFBP4, and IGFBP7 were determined by the Magnetic Luminex Assay kit (R&D Systems Europe, Ltd, Abingdon, UK) following the manufacturer's protocol.

### 2.5. Statistical Analysis

All descriptive statistics for continuous variables between people with and without OSA were done using Student's *t*-test and reported as mean ± standard error mean. A paired *t*-test was used to determine the significance of differences in means within each group between baseline and 3 months postsurgery. A chi-square test was used to compare the gender between people with and without OSA. Correlation between variables was calculated by Pearson correlation coefficient, with a *p* value <0.05 indicating statistical significance. Univariate and multivariate linear regression analysis was done for all population with and without OSA to predict the variables associated with IGFBP4. All statistical analysis was performed using SPSS for Windows version 25.0 (IBM SPSS Inc., USA).

## 3. Results

### 3.1. Study Population Characteristics

Table 1 summarizes the general characteristics of our study population. The study groups were classified into control and OSA groups that were age and BMI matched. Control participants had a mean age of 5 year, while participants with OSA had a mean age of 43 ± 2 years (*p* = 0.246). The mean BMI in the control group was 28.59 ± 0.99 kg/m^2^, while OSA participants had a BMI of 30.2 ± 0.63 kg/m^2^ (*p* = 0.181). There was no significant difference in parameters of the lipid profile comparing control group with the OSA group, and this included levels of total Chol, TG, and LDL with the exception of HDL where people with OSA showed a significantly reduced HDL levels 1.08 ± 0.04 mmol/L (*p* = 0.037) compared to the control group 1.25 ± 0.07 mmol/L (Table 1).

### 3.2. Polysomnography for OSA Diagnosis

The AHI score was used to diagnose people with OSA and classify patients into three subgroups according to OSA severity, i.e., mild, moderate, and severe. People with OSA had significantly higher OSA indices score compared to the control participants. This is demonstrated as higher apnea (AI), hypopnea (HI), and AHI scores in people with OSA compared to the control that is presented in Table 1.

### 3.3. Baseline Levels of Plasma IGFBPs

**Figure 1:**
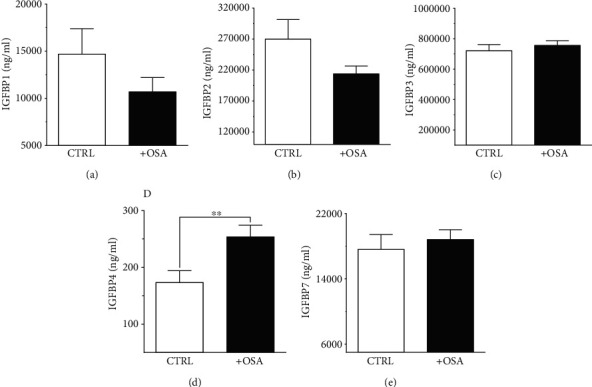
Comparing baseline levels of circulating IGFBPs in people with and without OSA. (a) IGFBP1 showed no significant difference (*p* = 0.203) between people with OSA (10695.42 ± 1510.3 ng/ml) and control (14687.01 ± 2692.49 ng/ml). (b) IGFBP2 levels were not significantly different (*p* = 0.139) comparing people with OSA (213922.55 ± 12632.2 ng/ml) and control (269837.02 ± 31643.27 ng/ml). (c) Levels of IGFBP3 are comparable between people with OSA (756438.94 ± 30196.72 ng/ml) and control (721032.69 ± 40291.55 ng/ml). (d) Circulating IGFBP4 was significantly higher (*p* = 0.008) in people with OSA (253.65 ± 20.46 ng/ml) compared to control (173.35 ± 20.86 ng/ml). (e) IGFBP7 levels showed no difference (*p* = 0.234) between OSA (18822.84 ± 1201.21 ng/ml) and control (17622.79 ± 1810.54 ng/ml).

Levels of plasma IGFBPs were different between people with OSA and control participants (Table 1). Although not significant, people with OSA showed lower level of IGFBP1 and IGFBP2 compared to their levels in the control group (Figure 1). There was a slight rise in IGFBP3 and IGFBP7 in people with OSA compared to the control group; however, the difference was insignificant. Levels of IGFBP4 were significantly higher in people with OSA, compared to the control group.

### 3.4. IGFBP4 Is an Independent Predictive Marker for People with OSA

Pearson's correlation analysis for people with OSA, without OSA, and all population combined was conducted to investigate the correlation between IGFBP4 and OSA indices (Table 2). We found a significant and positive correlation between the increase in IGFBP4 at baseline with AHI (*r* = .575, *p* < 0.001), AI (*r* = .703, *p* < 0.001), HbA1c (*r* = 0.501, *p* = 0.003), and WBC (*r* = 0.380, *p* = 0.026) and an inverse correlation with O_2_ saturation (*r* = −0.423, *p* = 0.033) and HDL (*r* = −0.378, *p* = 0.028) in people with OSA. Variables that were significantly correlated with IGFBP4 in OSA population and all population were used for multiple stepwise regression analysis model. Multiple stepwise regression analysis (Table 3) in OSA population showed that AHI scores (*β* = 0.575, *p* = .001), HbA1c% (*β* = 0.390, *p* = .007), and O_2_ saturation% (*β* = −0.417, *p* = .021) were significant predictors of IGFBP4 (*F*_1,40_ = 9.417, *p* = .006, and *r*^2^ = 22%). However, in all population (Table 3), IGFBP4 marker is a significant predictor with AHI scores (*F*_1,68_ = 6.493, *p* = .027, and *r*^2^ = 32%).

### 3.5. A Surgical Intervention Induced Improvement in AHI Score and IGFBP4 Levels

**Figure 2:**
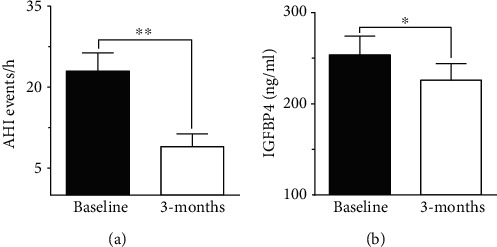
A significant improvement in AHI and IGFBP4 after 3 months of surgical intervention. (a) A significant decline in AHI index (10.7 ± 2.3 events/h, *p* = 0.013) after 3 months of surgery, reflecting a substantial improvement in OSA. (b) IGFBP4 showed a significant reduction (225.89 ± 18.16 ng/ml, *p* = 0.012) after 3 months of surgery.

To evaluate the effect of surgical intervention on OSA, we studied changes after 3 months of surgery. Patients demonstrated an improvement postsurgery, which was reflected by a significant improvement in AHI score (8.97 ± 2.3 events/h, *p* < 0.001, Figure 2). There was a decline in HI score after 3 months of surgery (6.63 ± 1.99 events/h) but the change was not significant compared to baseline score. In our study population, IGFBP4 was the only protein showing a significant reduction (225.89 ± 18.16 ng/ml, *p* = 0.012) after 3 months of surgery (Figure 2). The change in other IGFBPs after surgery was negligible (Table 4).

## 4. Discussion

OSA is increasingly becoming a major health problem that is aggravated with the global increase in incidence of obesity, diabetes, and CVD. In this study, we report a substantial rise in levels of circulating IGFBP4 in people with OSA compared to people without OSA. The rise in IGFBP4 correlated significantly and positively with OSA indices and both FBG and HbA1c in people with OSA. Multivariate linear regression analysis also presented IGFBP4 as a marker for people with OSA. Collectively, our data suggests a possible relationship between increased levels of IGFBP4 and OSA in the current study. Treating OSA with MLS concurred with a significant reduction in IGFBP4 levels, which further emphasized the link between IGFBP4 and OSA.

Obstructive sleep apnea is the most prevalent form of sleep-disordered breathing conditions affecting people with obesity. People with OSA experience intermittent hypoxia that is induced by repeated episodes of hypopnea and apnea. Intermittent hypoxia has been found to promote oxidative stress, systemic and vascular inflammation, and endothelial dysfunction [31]. It was also reported to cause gradual progression of daytime hypertension and the consequent long-term cardiovascular comorbidities. One of the systems affected by OSA is the GH/IGF axis. Due to its circadian rhythm, the activity of GH/IGF-I is influenced by disturbed sleep patterns [32]. Previous studies showed that people with obesity and OSA had an impaired GH/IGF-I axis function [32]; however, the effect of OSA on this axis was lost with increased age [33]. Ursavas et al. demonstrated that the presence of OSA per se was a risk factor for having low levels of IGF-I [18]. In a similar manner to the GH/IGF-I axis, IGFBPs are also governed by circadian regulatory mechanisms [34] and they contribute substantially to regulating the activity and bioavailability of IGF-I; thus, IGFBPs are expected to be influenced by OSA.

A characteristic of OSA is the cyclic episodes of complete or partial occlusion of the upper airway, leading to fragmented sleep, hypoxemia, and hypercapnia. The repeated events of arousal and desaturation during sleep cause an intermittent hypoxia/reoxygenation damage (IHR). Chronic hypoxia is a key feature of OSA, and it can lead to pathophysiological complications such as arterial hypertension [35], coronary artery disease [36], myocardial infarction [37], or other cardiovascular complications as atherosclerosis [38]. This is in addition to various metabolic and cognitive consequences and risk of cancer [37]. Previous studies reported increased expression of IGFBP1, IGFBP3 [39], and IGFBP4 [40] in response to hypoxia. However, the link between IGFBPs and OSA is not clear [33]. In our study, levels of circulating IGFBP3 did not differ between people with and without OSA (Table 1), and IGFBP3 showed no association with OSA indices (Table 3). This came in agreement with a previous report, which found no association between IGFBP1, IGFBP3, and measures of OSA [33]. Nonetheless, we found a significant increase in baseline levels of IGFBP4 in people with OSA compared to the control group (Figure 1). Performing corrective upper airway surgeries to treat OSA relieved the obstructed airflow that improved hypoxic conditions, and this might have contributed to the significant reduction in circulating IGFBP4 (Figure 2). Additionally, we found baseline IGFBP4 to be correlated with both FBG and HbA1c in people with OSA (Table 3), which implicated a potential link to glucose metabolism. Although IGFBP4 was not shown to contribute to glycemic control, other IGFBPs were found to play a role in glucose homeostasis [41]. At a state of obesity and insulin resistance, levels of fasting IGFBP1 were significantly reduced and this functioned as a predictive biomarker for developing abnormal glucose regulation [41]. Additionally, IGFBP2 was presented as a biomarker of insulin sensitivity and reduced levels of circulating IGFBP2 correlated with insulin resistance [41]. On the other hand, an overexpression of IGFBP3 concurred with impaired glucose tolerance [42, 43], and an increase in IGFBP3 was associated with a higher risk of T2D in women [44]. To the best of our knowledge, IGFBP4 was not previously linked to glycemic control indicators. Nonetheless, in a study by Jacot and Clemmons, high glucose levels were found to increase IGFBP4 proteolysis, which was proposed as a regulatory mechanism to preserve the IGF-I hypoglycemic effect, while insulin induced IGFBP4 mRNA expression and caused a substantial rise in its protein level [45].

Previous studies showed a positive correlation between IGFBP4 and age [28]. Nonetheless, in our study, the increase in IGFBP4 was age independent, and it was limited to people with OSA. The significant correlation with OSA indices suggested IGFBP4 as a potential diagnostic marker. This finding was supported by the logistic regression analysis (Table 3). Additionally, the link between IGFBP4 and OSA was further supported by the significant decline in IGFBP4 levels 3 months after MLS. The improvement in OSA status was reflected by an improved AHI score (Figure 2(a)) that was concomitant with a significant decline in levels of circulating IGFBP4 (Figure 2(b)). The link between OSA and elevated levels of IGFBP4 came in agreement with the upregulation of IGFBP4 expression in response to hypoxia [40]. According to Minchenko et al., we would speculate a concomitant rise in IGFBP4 and OSA severity due to increased hypoxia, which is reflected in our data. The effect of hypoxia on IGFBPs was previously reported with IGFBP7 in kidney disease. IGFBP7 is an acknowledged predictive biomarker of acute kidney injury [46] that demonstrates increased levels with conditions of hypoxia and atherosclerotic renal artery stenosis due to decreased renal blood flow [47]. Additionally, hypoxia was found to induce the expression of human IGFBP1 gene by activating the hypoxia-inducible factor-1 (HIF-1) pathway [48].

Currently, our understanding of the physiological significance of the increase in IGFBP4 level is limited. However, our data accentuated a potential role for IGFBP4 in OSA, where OSA-induced hypoxia might be playing a role in inducing a rise in circulating IGFBP4 levels. Although IGFBP4 appears as a potential biomarker for the diagnosis and prognosis of OSA, the functional significance of this association is not clear, and further investigation is required to elucidate the functional importance of IGFBP4 in OSA and its role (if any) in glycemic control. The main limitation of this study is the limited number of participants dictated by the nature of our study with surgery intervention.

## 5. Conclusions

In conclusion, we report a significant increase in circulating IGFBP4 in a group of adults with OSA, and we present IGFBP4 as an OSA biomarker. IGFBP4 correlated with OSA indices at baseline, which propounds a potential link between IGFBP4 and OSA or an OSA-related factor. Three months following a corrective intervention, there was a significant improvement in OSA, which concurred with a significant decline in circulating IGFBP4. Although the functional significance of this finding is obscure, it suggests IGFBP4 as a biomarker for OSA-induced hypoxia. The repeated episodes of hypoxia/reoxygenation would induce a chronic injury pathway, which might be counteracted by increased expression of IGFBP4.

## Figures and Tables

**Table 1:** Clinical and anthropometric characteristics of participants at baseline.

Variable	Non-OSA (*N* = 28)	OSA (*N* = 41)	
	Average ± SEM	Average ± SEM	*p* value
Age (years)	43.50 ± 2.03	40.41 ± 1.68	0.246
Gender	23 (82.1%)/5 (17.9%)	34 (82.9%)/7 (17.1%)	0.933
Weight (kg)	85.28 ± 3.51	89.06 ± 2.30	0.372
Height (cm)	172.36 ± 1.80	171.48 ± 1.13	0.682
BMI (kg/m^2^)	28.59 ± 0.99	30.18 ± 0.63	0.181
Pulse	73.21 ± 2.37	77.87 ± 1.74	0.119
SBP (mmHg)	125.04 ± 2.28	125.89 ± 2.18	0.786
DBP (mmHg)	74.75 ± 1.62	74.13 ± 1.66	0.790
ESS	2.58 ± 0.54	13.45 ± 1.30	<0.001^∗∗^
AHI (events/h)	2.20 ± 0.25	21.03 ± 2.54	<0.001^∗∗^
AI (events/h)	1.00 ± 0.19	4.23 ± 1.29	0.019^∗^
HI (events/h)	1.20 ± 0.25	17.25 ± 2.13	<0.001^∗∗^
T. Chol (mmol/L)	4.98 ± 0.19	5.09 ± 0.19	0.681
HDL (mmol/L)	1.25 ± 0.07	1.08 ± 0.04	0.037^∗^
LDL (mmol/L)	3.16 ± 0.18	3.33 ± 0.18	0.525
TG (mmol/L)	1.25 ± 0.11	1.51 ± 0.15	0.178
GLU (mmol/L)	5.85 ± 0.33	5.92 ± 0.20	0.864
HbA1c (%)	5.86 ± 0.23	5.84 ± 0.13	0.939
WBC (10^9^/L)	6.58 ± 0.39	7.07 ± 0.30	0.335
C-pep (pmol/L)	2514.62 ± 291.03	3633.65 ± 293.32	0.009^∗∗^
Insulin (U/L)	7.46 ± 0.91	10.93 ± 1.32	0.035^∗^
IGFBP1 (ng/ml)	14687.01 ± 2692.49	10695.42 ± 1510.3	0.203
IGFBP2 (ng/ml)	269837.02 ± 31643.27	213922.55 ± 12632.2	0.139
IGFBP3 (ng/ml)	721032.70 ± 40291.55	756438.94 ± 30196.72	0.485
IGFBP4 (ng/ml)	217.60 ± 21.75	289.74 ± 23.30	0.028^∗^
IGFBP7 (ng/ml)	20307.47 ± 1240.38	18822.84 ± 1201.21	0.400

Data are mean ± standard error mean; SBP: systolic blood pressure; DBP: diastolic blood pressure; ESS: Epworth Sleepiness Scale; AHI: apnea-hypopnea index; AI: apnea Index; HI: hypopnea index. ^∗^*p* < 05; ^∗∗^*p* < 01, indicating high statistical difference.

**Table 2:** Pearson correlation between IGFBP4 and various variables in all study groups.

Variable	Non-OSA		OSA		All populations	
	*r*	*p* value	*r*	*p* value	*r*	*p* value
Age (in years)	-0.208	0.330	0.106	0.552	-.052	.700
Weight (kg)	-0.114	0.594	0.376^∗^	0.029	.209	.116
Height (cm)	0.080	0.710	0.308	0.076	.200	.133
BMI (kg/m^2^)	-0.196	0.359	0.291	0.094	.142	.288
Pulse	0.024	0.913	0.117	0.522	.144	.288
SBP (mmHg)	0.014	0.949	0.195	0.285	.109	.423
DBP (mmHg)	-0.054	0.803	0.183	0.315	.066	.629
ESS	0.142	0.696	-0.092	0.727	.145	.472
AHI (events/h)	-0.296	0.170	0.575^∗∗^	0.0001	.522^∗∗^	.0001
AI (events/h)	0.027	0.903	0.703^∗∗^	0.0001	.649^∗∗^	.0001
HI (events/h)	-0.316	0.142	0.399	0.054	.458^∗∗^	.001
T. Chol (mmol/L)	-0.085	0.693	0.142	0.424	.089	.505
HDL (mmol/L)	0.024	0.911	-0.378^∗^	0.028	-.242	.067
LDL (mmol/L)	-0.101	0.638	0.152	0.398	.100	.459
TG (mmol/L)	-0.015	0.943	0.220	0.211	.185	.163
GLU (mmol/L)	-0.265	0.211	0.242	0.168	.047	.727
HbA1c (%)	-0.264	0.212	0.501^∗^	0.003	.169	.204
WBC (10^9^/L)	0.047	0.827	0.380^∗^	0.026	.310^∗^	.018
C-pep (pmol/L)	0.022	0.918	0.224	0.203	.241	.069
Insulin (U/L)	0.037	0.864	0.144	0.416	.182	.172
IGFBP1 (ng/ml)	0.040	0.763	0.060	0.736	.040	.763
IGFBP2 (ng/ml)	0.168	0.335	0.171	0.374	.168	.335
IGFBP3 (ng/ml)	-0.043	0.751	-0.116	0.514	-.043	.751
IGFBP7 (ng/ml)	0.037	0.832	0.127	0.512	.037	.832
O_2_ saturation	-0.089	0.616	-0.423^∗^	0.033	-.211	0.083

Pearson coefficient (*r*); ^∗^*p* < .05; ^∗∗^*p* < .01, indicating high statistical difference.

**Table 3:** Multivariate stepwise linear regression analysis for IGFBP4 predictors.

Variables	OSA group		All population	
	*β*	*p* value	*β*	*p* value
AHI	0.575	0.001	0.501	0.037
HbA1c	0.390	0.007	0.126	0.093
O_2_ saturation	-0.417	0.021	-0.091	0.299

The following variables were included: age, gender, BMI, ESS, WBC, insulin, AHI, O_2_ saturation, and HbA1c.

**Table 4:** Changes in clinical characteristics comparing baseline to 3 months postintervention.

Variable	Baseline (*N* = 41)	Post 3 months (*N* = 41)	
	Mean ± SEM	Mean ± SEM	*p* value
Weight (kg)	89.06 ± 2.30	86.46 ± 2.73	0.178
Height (cm)	171.48 ± 1.13	170.40 ± 1.64	0.490
BMI (kg/m^2^)	30.18 ± 0.63	29.71 ± 0.78	0.166
Pulse	77.87 ± 1.74	76.81 ± 2.41	1.000
SBP (mmHg)	125.89 ± 2.18	124.62 ± 2.86	0.518
DBP (mmHg)	74.13 ± 1.66	77.19 ± 2.19	0.411
ESS	13.45 ± 1.30	3.35 ± 0.58	<0.001^∗∗^
AHI (events/h)	21.03 ± 2.54	10.77 ± 2.37	0.006^∗∗^
AI (events/h)	4.23 ± 1.29	0.21 ± 0.14	0.008^∗∗^
HI (events/h)	17.25 ± 2.13	4.54 ± 1.74	0.004^∗∗^
T. Chol (mmol/L)	5.09 ± 0.19	5.01 ± 0.25	0.664
HDL (mmol/L)	1.08 ± 0.04	1.11 ± 0.05	0.839
LDL (mmol/L)	3.33 ± 0.18	3.27 ± 0.26	0.682
TG (mmol/L)	1.51 ± 0.15	1.40 ± 0.09	0.789
GLU (mmol/L)	5.92 ± 0.20	5.88 ± 0.23	0.711
HbA1c (%)	5.84 ± 0.13	5.63 ± 0.11	0.334
WBC (10^9^/L)	7.07 ± 0.30	6.21 ± 0.23	0.038^∗^
C-pep (pmol/L)	3633.65 ± 293.32	3202.32 ± 229.21	0.221
Insulin (U/L)	10.93 ± 1.32	9.49 ± 0.84	0.507
IGFBP1 (ng/ml)	10695.42 ± 1510.3	11407.10 ± 2218.27	0.678
IGFBP2 (ng/ml)	213922.55 ± 12632.2	201469.07 ± 14709.04	0.231
IGFBP3 (ng/ml)	756438.94 ± 30196.72	730163.84 ± 30757.08	0.435
IGFBP4 (ng/ml)	289.74 ± 23.30	249.13 ± 17.50	0.027^∗^
IGFBP7 (ng/ml)	18822.84 ± 1201.21	19315.96 ± 1199.77	0.439

Data are mean ± standard error mean; SBP: systolic blood pressure; DBP: diastolic blood pressure; ESS: Epworth Sleepiness Scale; AHI: apnea-hypopnea index; AI: apnea index; HI: hypopnea index. ^∗^*p* < .05; ^∗∗^*p* < .01, indicating high statistical difference.

## Data Availability

The datasets included in this study are not available for sharing from the corresponding authors due to unpublished data and ethical restrictions by the institute.
